# The Benefits of Increased Space and Habitat Complexity for the Welfare of Zoo-Housed King Penguins (*Aptenodytes patagonicus*)

**DOI:** 10.3390/ani13142312

**Published:** 2023-07-14

**Authors:** Grace Fuller, Megan Jones, Kylen N. Gartland, Sara Zalewski, Matthew R. Heintz, Stephanie Allard

**Affiliations:** 1 Center for Zoo and Aquarium Animal Welfare and Ethics, Detroit Zoological Society, Royal Oak, MI 48067, USAkgartland@dzs.org (K.N.G.);; 2 National Aquarium, Baltimore, MD 21202, USA; sallard@aqua.org

**Keywords:** exhibit design, animal welfare, post-occupancy evaluation, data-loggers, time–depth recorders, aquatic behavior

## Abstract

**Simple Summary:**

Habitat design influences every aspect of welfare for captive animals, including the sensory milieu, opportunities to forage for food, the ability to make choices about where to engage in species-typical behaviors, and the opportunity to regulate proximity to other animals sharing a space. Moving a group of animals from one designed space to another provides an opportunity to observe how their behavior is shaped by habitat design features. In this study, we observed the behavior of ten king penguins as they were transferred back and forth between two habitats at the Detroit Zoo. The Penguinarium, although state of the art for its time, opened in 1968 and offered less space and complexity than the naturalistic, expansive Polk Penguin Conservation Center (PPCC), which opened in 2016. These penguins spent more time swimming when they had access to the substantially larger pool of the PPCC. They also engaged in more positive social behaviors, such as species-typical displays and allopreening, and fewer aggressive behaviors in the PPCC. The results support a positive connection between the design of the PPCC and improved welfare for these king penguins.

**Abstract:**

Zoos and aquariums accredited by the Association of Zoos and Aquariums embrace animal welfare as a foundational principle of habitat design. Modern habitats are designed to provide animals with choices and agency over their environment, and to encourage species-appropriate behavior and space use. In 2016, the Detroit Zoological Society opened the Polk Penguin Conservation Center, a 3065.80 m^2^ facility that features a naturalistic design. The building was designed to optimize animal welfare by incorporating various substrates, nesting sites, and a 1234 kL pool with elements of underwater complexity. The facility houses a mixed-species group of penguins that were previously housed in a smaller habitat that opened in 1968. Between 2015 and 2022, we opportunistically monitored the behavior of ten king penguins (*Aptenodytes patagonicus*) as they moved back and forth between the two buildings while additional modifications were made to the new habitat. We collected 695 h of behavioral observations and 10,416 h of data from flipper-mounted time–depth recorders. We found that the king penguins spent less time engaged in aggression and more time engaged in swimming and positive social behaviors in the redesigned space. They also spent less time in proximity to other species of penguins and more time alone. These behavioral trends suggest that increased space and environmental complexity had positive welfare benefits for these penguins.

## 1. Introduction

Principles of zoo habitat design have coevolved with the growing body of knowledge on animal welfare. Until the development of modern ethology in the late 20th century, animal enclosures were designed to prioritize sanitation and visibility, resulting in largely barren environments [1]. Zoos have since embraced more naturalistic design schemes as a way to mitigate the adverse effects of captivity and promote species-typical behavior [2,3]. Naturalism is a heterogenous design concept that may incorporate aesthetic qualities of wild spaces, functionality in eliciting species-typical behavior, or both. Simply providing a large green space does not ensure an adequate degree of complexity to keep residents occupied [4]. Thus, enclosure naturalism does not necessarily indicate suitability for animal residents [5]. Animal welfare is defined as the animal’s subjective experience of four functional domains; the physical domain includes the enclosure, but welfare is also shaped by nutritional, health, and behavioral domains, all of which must be considered when evaluating an individual’s welfare [6].

Post-occupancy evaluations (POEs) harness a within-subjects design to directly evaluate enclosure suitability by comparing the same group of animals in different exhibit spaces [3]. The first zoo POEs noted dramatic changes in behavior associated with moving great apes from 20th-century “hard” zoo architecture to naturalistic enclosures, in which rates of agonistic and abnormal behaviors declined precipitously [3]. In spite of their great potential to inform exhibit design, POEs remain relatively rare in the scientific literature, with existing studies showing a taxonomic bias towards mammals [1]. However, increases in exhibit complexity and size have been shown to positively impact behavioral patterns in diverse taxa. A habitat renovation led to decreased repetitive behavior in Port Jackson sharks (*Heterodontus portusjacksoni*), but whether these changes were due to a visitor barrier or other enclosure improvements is unclear [7]. Madagascar giant hognose snakes (*Leioheterodon madagascariensis*) demonstrated increased behavioral diversity and investigative behaviors after their move to an enriched enclosure. In this case, it was not possible to determine whether the behavioral changes were due to the increase in exhibit size, complexity, or both. It was also difficult to disentangle these factors during a POE conducted on Galapagos tortoises (*Chelonoidis* spp.) at the ZSL London Zoo [8]. As these examples illustrate, it can be difficult, if not impossible, to identify specific causal factors for changes in animal behavior in most POE studies, given the multitude of factors that are generally altered in habitat improvements and the entangled relationship between space and complexity.

Despite the popularity and charismatic nature of penguins, studies of their welfare were rare until recent years, with existing research focusing primarily on visitor effects [9,10,11,12,13,14,15,16]. Pool use has been a primary focus of other studies. Pool use in Humboldt penguins (*Spheniscus humboldti*) was correlated with available land space across European zoos [17], whereas Magellanic penguins (*S. magellanicus*) spent more time swimming after their pool was changed from freshwater to saltwater [18]. Providing zoo-housed Humboldt penguins with a mock whale skeleton also increased the amount of time they spent swimming [19]. Finally, pool use by a group of gentoo penguins (*Pygoscelis papua*) was correlated with higher visitor numbers, as was the overall behavioral diversity expressed by the penguin group [20].

Pool use may indicate a better welfare state for captive penguins. Magellanic penguins that spend more time in water have a reduced incidence of pododermatitis (commonly known as bumblefoot) [21], whereas hatching success for Humboldt penguins increased with greater pool size in a survey of British zoos [22]. Using pressure sensors (time–depth recorders; TDRs), another study found that macaroni (*Eudyptes chrysolophus*) and southern rockhopper (*E. chrysocome*) penguins increased their time spent swimming, as well as their overall habitat use, after receiving phacoemulsification surgery for cataract removal [23].

Penguins can be difficult to track underwater in captive settings, but these challenges are even greater for field researchers. To understand the behavioral ecology of seabirds that spend much of their life foraging in the open ocean, researchers must utilize TDRs or other animal-attached devices [24]. King penguins (*Aptenodytes patagonicus*) are the second-largest penguin species [25]. Based on TDR data, they are known to forage at average depths of 120–150 m (but up to 300 m) as they hunt for their preferred prey, lantern fish (*Myctophidae*) [26]. The duration and length of foraging trips depend on the stage of the 14–16-month breeding cycle. While incubating and brooding chicks, king penguins in the Crozet archipelago foraged around 400 km from the colony; their foraging range increased over the winter as adults left chicks in créches, traveling thousands of kilometers to forage in Antarctic waters [27,28].

Although automated logging devices have been informative about the behavior of king penguins at sea, studies on land have revealed elements of their natural history related to breeding and molt. Penguins are unusual in that they undergo an annual catastrophic molt in which they replace all their feathers at the same time. King penguin molt occurs prior to the breeding season and can last from 13 to 39 days [25]. During this time, they are unable to forage for food, resulting in a fasting period during which they lose around half their body weight [25,29]. After a few weeks of post-molt foraging, the breeding season begins. King penguins lay eggs over a four-month period at the start of the austral summer, and chicks hatch after a 55-day incubation [30]. Chicks that hatch later in the season often do not survive the period of fasting that occurs during the winter créche period [30]. Pairs aggressively defend a breeding territory, likely because breeding success is much higher for pairs nesting towards the center of the colony [31]. Captive breeding success has generally been high for king penguins, although most chicks have been produced at a limited number of institutions. As of 2019, facilities accredited by the Association of Zoos and Aquariums (AZA) housed 287 king penguins in 16 facilities [32], including 14 individuals residing in a mixed-species colony at the Detroit Zoo.

The Detroit Zoological Society (DZS) opened the Polk Penguin Conservation Center (PPCC) in 2016. The building was designed with the goal of incorporating a more naturalistic design and increasing the size and complexity of the pool and land areas compared to the previous habitat at the Detroit Zoo, the Penguinarium. We conducted a POE of ten king penguins in this colony using a combination of live behavioral observations and automated monitoring of swimming behavior with TDRs. Given the strongly seasonal nature of king penguin behavioral ecology, we conducted observations over a multi-year period. We hypothesized that the greater size and complexity of the new habitat would be associated with positive changes in welfare indicators, including increases in time spent swimming, overall activity, and positive social behaviors.

## 2. Materials and Methods

### 2.1. Study and Habitat (Exhibit) Design

This study was conducted at the Detroit Zoo in Royal Oak, Michigan, U.S.A., and was approved by the DZS’s Animal Welfare and Management Committee. We collected the data presented here during two phases (Phase 1: 2015–2017 and Phase 2: 2019–2022) conducted over an eight-year period as the king penguins were transferred back and forth between two habitats (Table 1). We began data collection in 2015, when the penguins resided in the Zoo’s Penguinarium, and continued through their 2016 move to the PPCC until 2017. The PPCC opened to the public in April 2016; however, the king penguins were not moved into the habitat until May 2016, after they had completed their annual molt. In 2019, the PPCC was closed for additional waterproofing construction, so we monitored the penguins again through their move to the Penguinarium and subsequent return to the PPCC. The king penguins were gradually returned to the PPCC over a three-week period in June 2021 as they finished their annual molt. The PPCC reopened to the public on 14 February 2022. Buildings were open to the public during all data collection periods, with the exception of Penguinarium IV and the first seven months of PPCC V (Table 1).

**Figure 1:**
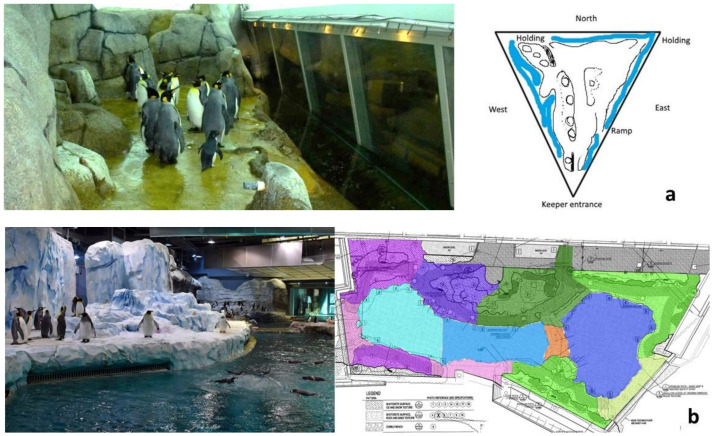
Penguinarium (**a**) and Polk Penguin Conservation Center (PPCC) (**b**) habitats. The Penguinarium photo shows the east side of the triangular habitat, which is where the king penguins spent most of their time. The PPCC photo shows the Antarctic side of the habitat, where the king penguins congregated, corresponding to the top left region (dark pink and purple) on the habitat blueprint. Photos courtesy of the Detroit Zoological Society.

The Penguinarium opened in 1968 and features a ring-shaped freshwater pool 1.8 m deep containing approximately 132.5 kL of water. The habitat is roughly triangular, with a central portion that can be blocked off to separate birds or opened to create more space during the breeding season (Figure 1a). The land portion of the habitat features gunite rockwork and pathways. Gunite areas accessible to the penguins reach about 2 m height in some places, and rockwork occupies more land space on the west and north sides of the habitat. The king penguins tend to prefer the east side of the habitat, in which gunite occupies minimal land space. Air and water temperature in the Penguinarium have minor seasonal fluctuations based on external conditions, with air temperature in the habitat ranging from 7 to 9 °C and water temperature from 7 to 10 °C. Fluorescent fixtures provide lighting that is seasonally varied to create a northern hemisphere (Arctic) light cycle, with the shortest day of the year coinciding with the local light cycle in Detroit. The average daytime irradiance in the habitat is 0.24 ± 0.04 (SE) W/m^2^. Habitat modifications were made to the Penguinarium in 2019 prior to the Penguinarium IV study period. Specifically, rockwork on the north side was reduced to open up the central portion of the habitat, creating more space for the penguins. An ice machine was also installed to create fine ice or “snow” in the central portion of the habitat.

The public exterior of the PPCC was designed to resemble a tabular iceberg in the process of calving, with a waterfall running through the center of the structure adjacent to a visitor splash pad. The PPCC indoor penguin habitat features a 1234 kL freshwater pool with a depth of 7.6 m. The pool has an open design (Figure 1b) that includes an artificial kelp forest and simulated wave motion. A channel circles the back of the habitat, allowing the penguins to swim continuously without ever having to reverse direction unless they choose to do so. Water and air temperatures are kept at a steady 5.5 °C year round. Like the Penguinarium, the PPCC is maintained on an Arctic photoperiod. Light is provided by a mixture of metal halide and LED spotlights, with an overall average irradiance across the habitat of 0.99 ± 0.17 W/m^2^ on a sunny day. Additionally, UV-penetrable skylights cover a large portion of the habitat to provide natural light.

The PPCC habitat is divided into roughly two equal parts, with rockwork and theming on the southern portion that mimics a South American vista. This portion of the habitat includes resin and gunite pathways, as well as a portion paved in cobblestones. A submerged land bridge with a depth of ~20 cm extends across the main habitat pool to link the east and west sides of the habitat. A resin pathway runs along the perimeter of the habitat through submerged land (~18 cm deep) in front of the main viewing window. Gunite rockwork reaches heights exceeding 2 m in some places, with lower “troughs” running along the habitat border that can be filled with small rocks for nest building during the breeding season. Additional nesting areas are available at heights over 2 m in the central portion of the habitat. Small “waterfalls” create showers in several habitat locations to encourage bathing behavior. The northern portion of the habitat is designed as an Antarctic vista, with a resin pathway running around the perimeter, submerged land near the primary viewing window, similar gunite structures, and an ice machine used to create “snow”. Additionally, Dri-Dek was added throughout the habitat to promote foot health. Some modifications were made to the PPCC during the second construction period (between study phases), including widening the ramp from the central area of the habitat that leads into the pool and other substrate changes, including the addition of embedded rocks to the submerged land areas near the primary viewing windows and resurfacing deck areas. A second, smaller ice machine that was previously located next to the primary unit was also moved to the corner of the Antarctic land area.

### 2.2. Subjects and Husbandry

At the beginning of the study in 2015, the penguins lived in the Penguinarium in a mixed flock of 64 penguins representing four species: 8.6 (number male. number female) king, 2.1 gentoo (*P.p. ellsworthi*), 9.17 macaroni, and 6.15 southern rockhopper penguins. A group of 10.10 *P.p. papua* was added to the flock in 2016 after the Phase 1 move to the PPCC, bringing the total colony up to approximately 80 individuals. Four king penguins died during the study. Only one of these, King 26, was a subject, and she died of aspergillosis during a period when data were not being collected. The other deaths were attributed to chronic renal disease, hepatitis, and an infected carpal joint. All were individual-specific, rather than reflecting broader patterns of disease in the colony. Five king penguins, including two study subjects (Arthur and Gertie; Table 2), were transferred to or hatched at Detroit during the study as well. On 27 January 2020, 2.2 chinstrap penguins (*Pygoscelis antarcticus*) joined the flock in the Penguinarium, eventually moving back to the PPCC with all the penguins.

We observed a total of 10 king penguins over the entire study (Table 2). Eight individuals were included in the first phase of data collection from 2015 to 2017. Six individuals were observed during the second phase of data collection from 2019 to 2022. Four individuals were observed during both phases. The overall sample included five males and five females, with an average age of 14.73 ± 9.05 (SD) years based on the date when data collection started on that individual.

Penguin management was relatively consistent throughout the study. In the Penguinarium, the king penguins were sometimes given access to the center section to reduce breeding-related agonistic encounters. This was not necessary in the PPCC. In both habitats, their primary diet consisted of herring, which was provided in a variety of ways, including food pans, broadcast feeding in the pool, feeding in enrichment devices, and hand feeding. Each king penguin was hand-fed once or twice a day, receiving 2–6 herring per day on average. They also received capelin and silversides at some feeding events. Each king penguin also received one large bird supplement per day (5TLB, Mazuri, St. Louis, MO, USA). Keepers entered each habitat multiple times per day, with the primary cleaning period occurring in the morning before the handfeeding, which took place between 10:00 and 11:00. In the PPCC, divers cleaned the pool an average of 3–4 times per week. Penguins were sometimes moved off habitat to incubate eggs, and this impacted observational and TDR data collection for one female subject (King 26; see section on TDR data collection).

### 2.3. Observational Data Collection

We collected observational data on focal penguins during each study phase using instantaneous scan sampling of activity, substrate, social proximity, habitat area, and location on a map (in the PPCC only) at one-minute intervals. Observers also recorded all occurrences of brief event behaviors [33]. As a rule, observers recorded repeated all-occurrence events of the same behavior when at least five seconds elapsed between the events. The ethogram of behaviors used in this study is listed in Table 3.

**Figure 2:**
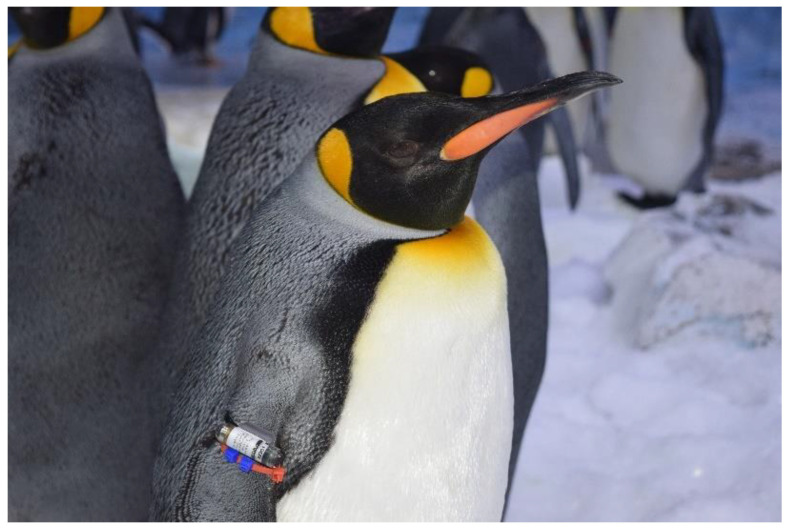
Penguin “King 77” wearing the time–depth recorder affixed to a flipper band using a custom-designed cradle (Lotek Wireless, Inc., Newmarket, ON, Canada). Photo courtesy of Lindsay Ireland.

Observers individually identified penguins by the colored beads on their flipper bands (Figure 2) or sometimes by a second, uniquely colored band. Observers did not individually identify social partners when scoring social proximity or modifiers for social interactions; instead, they marked the species involved in the encounter. The activity channel was mutually exclusive, meaning that the observer could only score one value in the channel on each interval. For social proximity, observers could score the focal penguin in proximity to any of the other species simultaneously. A penguin was considered in proximity to another individual if any part of them was within 0.3 m of another penguin, including outstretched flippers. Observers could also score proximity as alone or unclear.

Observers recorded data on paper check sheets or using the ZooMonitor program [34] on tablets (iPad Air (MD785LL/B) and iPad Air 2 (MNV72LL/A), Apple Inc., Cupertino, CA, USA). Each observation was ten minutes in duration for the 2015–2017 phase, whereas observations lasted five minutes in the 2019–2022 phase. All observations were balanced across two-hour time periods between 08:00 and 16:00. During two-hour shifts, observers recorded data on a list of randomly selected focal penguins; potential focal subjects included other species as well, although only king penguin data are reported here. Observers recorded data on the penguins in the order they were listed to avoid any selection bias for observing birds more readily visible or engaged in more conspicuous behaviors. If the behavior of the penguin was not visible for more than five (in 2015–2017) or three (in 2019–2022) intervals, the observer conducted a second observation during the same two-hour period. Only observations with a minimum of 50% behavioral visibility were considered sufficient for inclusion in analyses.

A total of 40 observers collected data for this study. Each observer passed a three-part reliability test, which included an identification test for all focal penguins, a multiple-choice test covering the ethogram, and three observations scored in tandem with one of the principal investigators. All observers maintained reliability above 90% based on the mean percentage difference calculated on tandem observations, and this criterion was reassessed quarterly for all observers.

### 2.4. Time–Depth Recorders

Data on pool usage were recorded using TDRs during the 2015–2017 phase. The TDR used in this study was the LAT1800L (Lotek Wireless, Inc., Newmarket, ON, Canada). The TDRs were affixed to cradles custom designed by the manufacturer using epoxy and secured to the penguins’ flippers using cable ties, which the penguins were accustomed to wearing for identification purposes (Figure 2). The LAT1800L TDR is approximately cylindrical in shape and measures 36 × 13 × 10 mm. The weight of the TDR in air with the cradle is 14.1 g, which was less than 0.2% of the body weight of the smallest king penguin in the study. We have previously demonstrated that wearing the TDRs coincided with only minor changes in the behavior of the penguins, with no welfare concerns [35].

Each penguin wore a TDR four times (twice in the Penguinarium and twice in the PPCC), with each period lasting approximately two weeks. We rotated seven TDR units among all the study subjects. Sometimes caretakers could not remove devices from penguins who stayed in the pool, so those penguins wore the TDRs for extra days. The mean duration wearing TDRs per deployment for the king penguins was 16.20 ± 1.24 (SD) days, with a range of 15–20 days. In each habitat, one of the sessions occurred roughly during the breeding season (June to August for king penguins), whereas the other occurred during the non-breeding season (October to February). The penguins did not wear TDRs during their annual molt. The exact dates that an individual penguin wore a TDR were matched between the two habitats. One king penguin (King 26) who incubated an egg during the study period only wore a TDR three times: twice in the Penguinarium and once in the PPCC. One penguin (King 77) wore a logger five times due to a device malfunction.

We also conducted a series of validation tests to ensure that the TDRs reliably recorded depth and wet/dry state in these freshwater penguin habitats [35]. We programmed the TDRs to record pressure and wet/dry state every six seconds for 24 h per day. Each two-week deployment resulted in 201,600 total data points, and when penguins wore the loggers for extra days, the additional data were discarded to keep analyses consistent between individuals. We downloaded data from the TDRs using Tag Talk (Lotek Wireless, Inc., Newmarket, ON, Canada) and exported them into Excel (v. 2016, Microsoft Corporation, Redmond, WA, USA) for further analysis.

### 2.5. Data Analysis

The total amount of TDR data for analysis represented 10,416 h of continuous data collection. Using the TDR data, we calculated the average total percentage of time spent swimming in each habitat. We also converted the raw pressure data to depth using the formula 1 dBar = 1.02 m. Using these data, we calculated the average proportion of time spent swimming at depths of 0 m (surface swimming), >0 to <2 m, >2 to <4 m, >4 to <6 m, and >6–8 m, which was the maximum depth in the PPCC. As with the time spent swimming, we calculated depth dispersion for each habitat.

The total amount of behavioral data analyzed here represents 694.8 h (5368 observations): 311.9 h (2554 observations) in the Penguinarium and 382.9 h (2814 observations) in the PPCC. Using the behavioral observations, we identified a subset of interval, all-occurrence, and proximity behaviors of interest as potential welfare indicators. Interval behaviors included swimming (surface swimming and diving combined), laying, feeding, and walking. Interval behaviors that comprised less than 1.00% of total observed behaviors were not considered sufficient for analysis. All-occurrence behaviors included rates of bathing (land and water combined), allopreening (giving and receiving), displays (ecstatic and mutual), total agonism (giving and receiving contact aggression and giving and receiving noncontact aggression combined), agonism given (contact and noncontact combined), and agonism received (contact and noncontact combined). Proximity behaviors included percentage of time in proximity to another king penguin, alone, or in proximity to an individual from another species. For each interval behavior, we calculated daily percentages corrected for total visibility. Visibility was assessed by conducting a daily calculation of how many scans were visible out of the total collected scans. All-occurrence behavioral rates were calculated based on the total number of visible observation minutes per day.

Following these initial descriptive analyses, all further analyses were conducted using SAS©, 9.4.1 (Cary, NC, USA). After conducting Kolmogorov–Smirnov tests for normality through the UNIVARIATE procedure, we found that all outcome variables of interest were non-normally distributed. Before beginning advanced inferential statistics, we confirmed that none of the variables (either outcome or predictors) were multicollinear. Given the non-normality of the outcome variables, we elected to perform non-parametric analyses. We used the NPAR1WAY procedure to run Wilcoxon two-sample tests using a Monte Carlo sampling method at 10,000 permutations to generate the test statistic and correct for the small sample size following a previously established methodology [36,37,38]. Only results that had an adjusted Monte Carlo significance (Pr ≥ |S-Mean|) of *p* < 0.05 were considered significant. Results with an adjusted Monte Carlo significance (Pr ≥ |S-Mean|) of 0.05 < *p* ≤ 0.1 were considered to be trending towards significance. The sample size reported is the number of individuals or the number of hours for individual results. Given that the study occurred in two distinct phases, we ran the Wilcoxon two-sample tests on both the total combined dataset and the data for phases 1 and 2 separately to ensure coherence. However, we elected to visually present the results with both phases combined given the high level of consistency of the results between phases.

Although habitat was our primary predictor variable of interest, we wanted to confirm that other confounding variables were not better predictors of differences in observed behavior. As such, we elected to run generalized linear mixed models (GLMMs) on counts of behaviors using the GLIMMIX procedure. GLMMs were run with a negative binomial distribution with an added Newton–Raphson ridging optimization technique and a maximum iteration limit of 100. The default optimization for GLIMMIX in SAS is a quasi-Newton algorithm. However, when working with over-dispersed data such as those found in many zoo-based studies and datasets with negative binomial distributions, the ridge-stabilized Newton–Raphson algorithm is a more appropriate option for optimizing different line-search methods and convergence criteria. Initial versions of all models included sex, age, time of day, season (breeding, molting, other), and habitat (Penguinarium or PPCC) as predictor variables. Although data were collected in two distinct phases (see Table 1), the consistency in trends across phases as established by the Wilcoxon tests did not necessitate the inclusion of “phase” as a predictor variable in all GLMMs. Additionally, “individual” was included as the random intercept, and the log of the total visibility for each observation was included as an offset variable. Final reported models include only significant independent variables. We used parameter estimates and associated t-tests to determine differences among levels of categorial variables and directional effects for continuous predictors. The only behavior not analyzed through a GLMM was allopreening due to limitations in available data.

## 3. Results

### 3.1. Time–Depth Recorder Data

**Figure 3:**
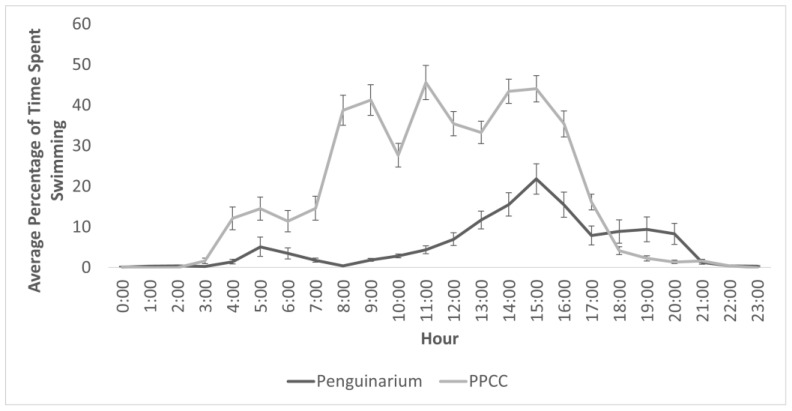
Percentage of time (mean ± SE) king penguins (N = 8) spent swimming for each hour of the day in the Penguinarium and Polk Penguin Conservation Center (PPCC), measured using time-depth recorders.

Data from the TDRs indicated an approximately threefold increase in time spent swimming in the PPCC compared to in the Penguinarium (Table 4 and Figure 3). Data collected from the TDRs indicated an increase in swimming between habitats across all individuals, with an overall range of 0.03 ± 0.00% (Lola) to 18.03 ± 6.65% (King 26) of time in the Penguinarium and 0.05 ± 0.02% (Lola) to 34.59 ± 11.41% (King 25) of time in the PPCC. Peak swimming time in the Penguinarium occurred at 15:00, whereas peak pool use in the PPCC occurred at 11:00, followed by 15:00 and 14:00 (Figure 3).

**Figure 4:**
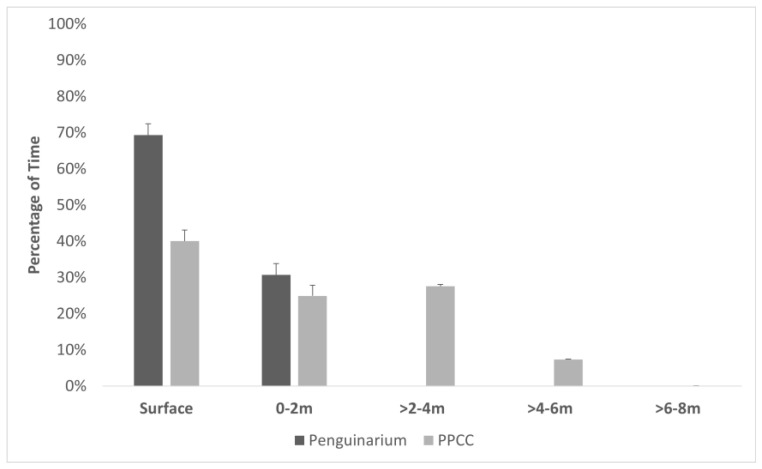
Percentage of time (mean ± SE) king penguins (N = 8) spent in five depth categories in the pools of the Penguinarium and Polk Penguin Conservation Center (PPCC), measured using time–depth recorders. Note that the pool in the Penguinarium is 2 m deep, whereas the pool in the PPCC is 8 m deep.

Based on TDR data, the penguins spent more time swimming at the surface in the Penguinarium than in the PPCC (Table 4; Figure 4). Time spent swimming at depths of 0–2 m did not significantly differ between the two habitats (Table 4). When they had access to the deeper pool in the PPCC, the penguins spent the most time swimming at depths of 2–4 m (Table 4). On average, the penguins swam significantly more deeply in the PPCC compared to in the Penguinarium (Table 4).

### 3.2. Behavioral Comparison across Study Phases

Despite variation in the amount of data between study phases and the length of time that elapsed between study phases, analysis demonstrated that behavioral trends were largely consistent across study phases (Table 5). Specifically, the percentage of time spent swimming, feeding, and laying was significantly higher in the PPCC compared to the Penguinarium across both phases (Table 5). The percentage of time spent walking was significantly higher in the Penguinarium than in the PPCC across both phases (Table 5). All-occurrence rates of agonism (given, received, and total) and bathing were higher in the Penguinarium than in the PPCC across both phases (Table 5). The percentage of time spent in proximity to another king penguin did not vary between habitats across both phases (Table 5). The all-occurrence rate of allopreening was significantly higher in the PPCC in Phase 1, but the difference between habitats was non-significant in Phase 2 (Table 5). The percentage of time spent alone was significantly higher in the Penguinarium in Phase 1, but the difference between habitats was non-significant in Phase 2 (Table 5). The percentage of time spent in proximity to a penguin from another species did not vary between habitats in Phase 1 but was significantly higher in the Penguinarium in Phase 2 (Table 5). The all-occurrence rate of displays was the only behavior to show conflicting results, with significantly higher rates in the PPCC in Phase 1 but significantly higher rates in the Penguinarium in Phase 2 (Table 5).

When analyses were run on the total dataset combining both study phases, most overall trends remained consistent (Table 6). However, feeding behavior was no longer significant and rather trended towards significance, and proximity to another king penguin became significant (Table 6). Specific results by behavior are discussed below.

### 3.3. Aquatic Behaviors

**Figure 5:**
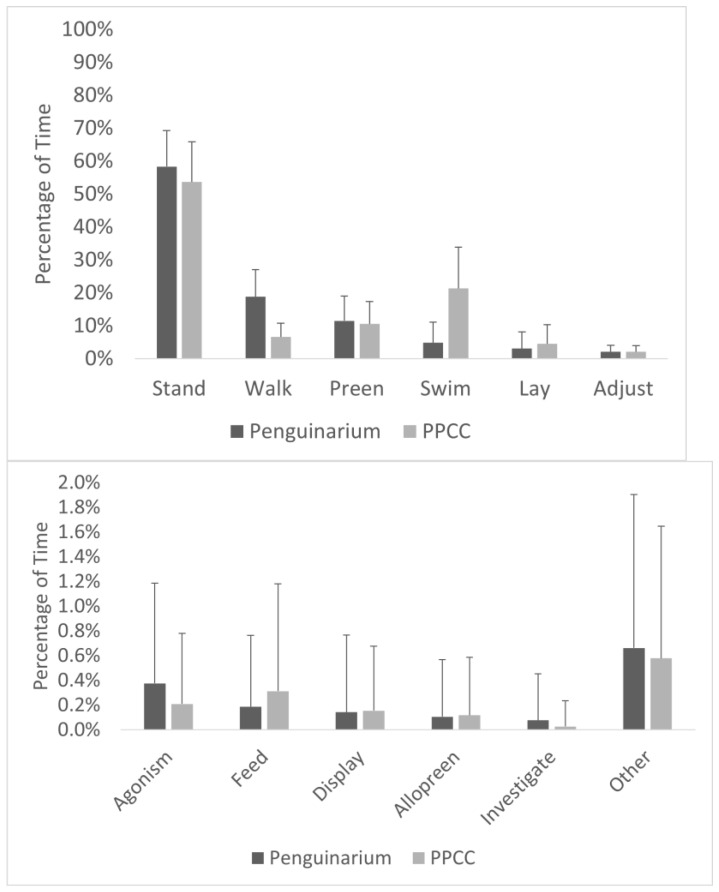
Percentage of visible time (mean ± SE) king penguins (N = 10) spent performing each behavior in their activity budget in the Penguinarium and in the Polk Penguin Conservation Center (PPCC). Behaviors are presented on two different scales to highlight rarer behaviors (bottom) and are combined for the two study phases.

**Figure 6:**
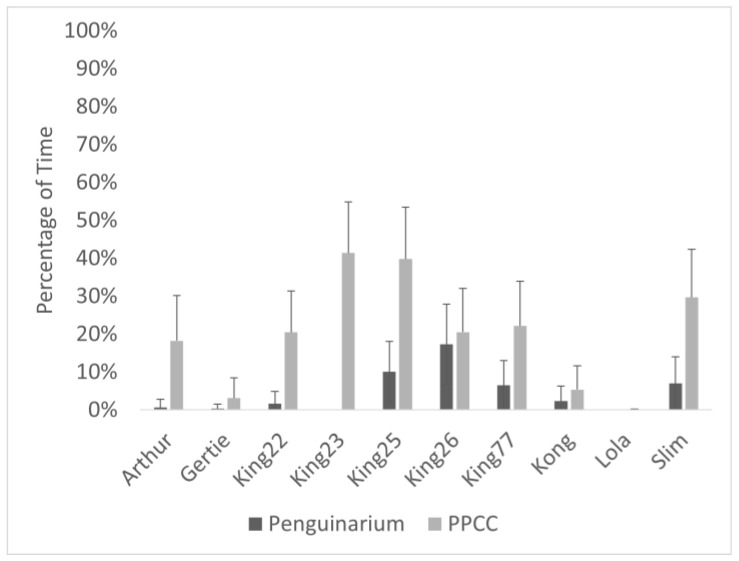
Percentage of visible time (mean ± SE) king penguins (N = 10) spent swimming in the Penguinarium and in the Polk Penguin Conservation Center (PPCC). Data are combined for the two study phases. Standard error bars are based on the number of months of data.

Observational data demonstrated high agreement with data collected through the TDRs. As a group, the king penguins spent significantly more time swimming when housed in the PPCC compared to in the Penguinarium (Table 6; Figure 5). At the individual level, average time spent swimming during the day ranged from 0.00 ± 0.00% of visible time (Lola) to 17.30 ± 10.59% of visible time (King 26) in the Penguinarium, whereas the range in the PPCC was from 0.04 ± 0.16% (Lola) to 41.34 ± 12.06% (King 23) (Figure 6). Although individual increases in time spent swimming between the Penguinarium and the PPCC varied in significance, every individual demonstrated some degree of increase. The GLMMs further demonstrated that swimming increased in the PPCC across all seasons but decreased during the molting season regardless of habitat (Table 7).

**Figure 7:**
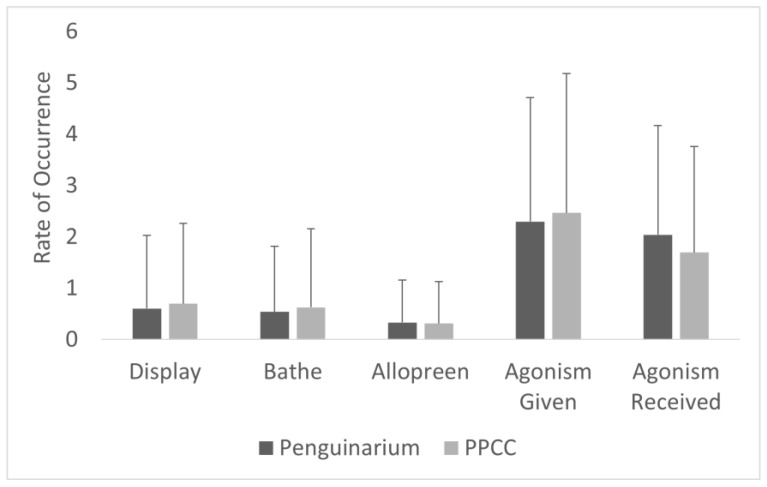
Rate per hour (mean ± SE) of all-occurrence event behaviors displayed by king penguins (N = 10) in the Penguinarium and in the Polk Penguin Conservation Center (PPCC). Data are combined for the two study phases.

All-occurrence instances of bathing per hour were significantly higher in the PPCC compared to in the Penguinarium (Table 6; Figure 7). Average instances of bathing per hour by individual ranged from 0.00 ± 0.00 (Gertie, King 23, King 26, King 77, Kong, Lola, Slim) to 0.50 ± 0.17 (King 25) in the Penguinarium and from 0.00 ± 0.00 (Lola) to 2.39 ± 0.80 (Arthur) in the PPCC.

### 3.4. Other Solitary Behaviors

Laying, feeding, and walking were the only solitary interval behaviors with sufficient data for analysis. The king penguins spent significantly more time laying in the PPCC compared to in the Penguinarium (Table 6; Figure 5). At the individual level, the percentage of time spent laying ranged from 0.00 ± 0.00% (King 23) to 9.53 ± 5.78% (King 77) in the Penguinarium and from 0.22 ± 0.92% (Slim) to 8.76 ± 4.95% (King 25) in the PPCC. The percentage of time spent feeding trended towards being significantly higher in the PPCC compared to in the Penguinarium (Table 6; Figure 5). At the individual level, feeding ranged from 0.00 ± 0.00% (Slim and King 22) to 0.56 ± 0.87% (Lola) in the Penguinarium and from 0.09 ± 0.27% (Slim) to 0.79 ± 1.59% (Arthur) in the PPCC. The percentage of time spent walking was significantly higher on average in the Penguinarium than in the PPCC (Table 6; Figure 5). Average time spent walking by individual ranged from 7.44 ± 2.59% (Kong) to 30.08 ± 8.65% (Gertie) in the Penguinarium and from 4.10 ± 1.80% (Kong) to 8.94 ± 3.53% (King 26) in the PPCC. The GLMMs demonstrated that feeding, laying, and walking were all significantly influenced by habitat. Specifically, laying and feeding increased in the PPCC, whereas walking decreased in the PPCC (Table 7). Laying additionally was generally lower in the mornings (A.M.) compared to in the afternoons (P.M.), feeding was higher among females compared to males, and walking was generally higher in the breeding season (Table 7).

### 3.5. Social Behaviors

Rates of all-occurrence social behaviors, including both positive social behaviors (allopreening and displays) and negative interactions (agonism), varied significantly between the two habitats (Table 6; Figure 7). The average rate of allopreening was significantly higher in the PPCC compared to in the Penguinarium. Instances of allopreening per hour ranged from 0.00 ± 0.00 (King 77) to 0.56 ± 0.92 (Arthur) in the Penguinarium and from 0.01 ± 0.07 (King 25) to 0.67 ± 1.16 (King 26) in the PPCC. The average rate of displays per hour was also significantly higher in the PPCC compared to in the Penguinarium (Table 6; Figure 7). Instances per hour ranged from 0.10 ± 0.09 (Slim) to 1.39 ± 0.25 (Kong) in the Penguinarium and from 0.16 ± 0.11 (Lola) to 1.63 ± 0.35 (King 22) in the PPCC. However, a GLMM demonstrated that rate of displays was more significantly influenced by season (higher in breeding season) and sex (higher among females than males) and only trended towards being significantly influenced by habitat (Table 7).

The average rate of given agonism, received agonism, and total agonism were all significantly higher in the Penguinarium (Table 6; Figure 7). Total instances of agonism per hour ranged from 2.70 ± 3.13 (Kong) to 13.04 ± 6.67 (King 23) in the Penguinarium and from 1.29 ± 1.02 (King 25) to 5.79 ± 4.32 (Arthur) in the PPCC. The GLMM demonstrated that habitat, season, and time of day were all variables that significantly influenced rates of all-occurrence agonism (Table 7). Specifically, according to the GLMM, the rate of agonistic interactions significantly decreased in the PPCC and tended to increase during the breeding season but decrease in the molting season (Table 7).

### 3.6. Social Proximity

**Figure 8:**
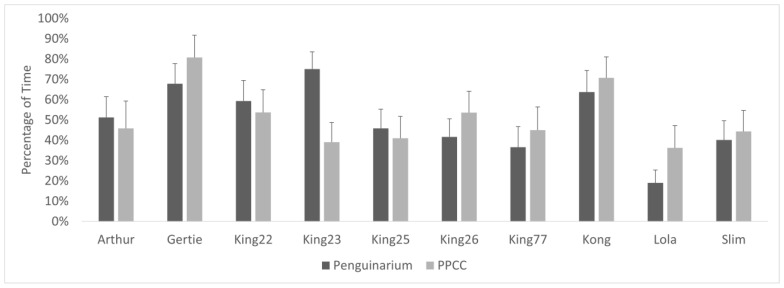
Percentage of visible time (mean ± SE) king penguins (N = 10) spent in proximity (within 0.3 m) to other king penguins in the Penguinarium and in the Polk Penguin Conservation Center (PPCC). Data are combined for the two study phases for individuals that participated in both. Standard error bars are based on the number of months of data.

**Figure 9:**
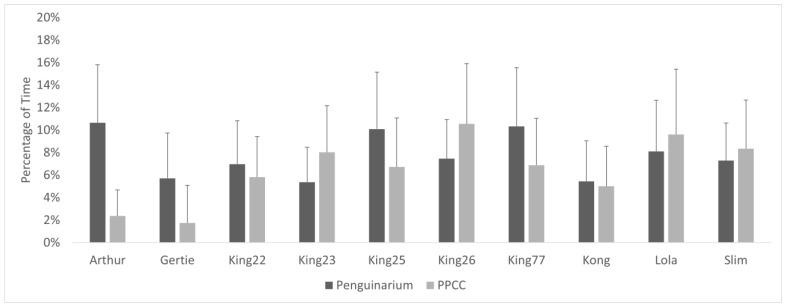
Percentage of visible time (mean ± SE) king penguins (N = 10) spent in proximity (within 0.3 m) to penguins of other species in the Penguinarium and in the Polk Penguin Conservation Center (PPCC). Data are combined for the two study phases. Standard error bars are based on the number of months of data.

When housed in the PPCC, the king penguins spent significantly less time in proximity to individuals from other species and significantly more time in proximity to other king penguins or alone (Table 6). This is not reflected in overall group averages and standard error due to highly mixed individual results. At the individual level, six individuals demonstrated increased proximity to other king penguins when in the PPCC, but four demonstrated decreased proximity (Figure 8). Similarly, six individuals displayed decreased proximity to penguins from other species while four demonstrated increased proximity (Figure 9). The GLMMs demonstrated that habitat was a significant influencing variable for all proximity measures (Table 7). However, age and season also had significant influences over proximity patterns.

## 4. Discussion

Overall, the king penguins in this study demonstrated increases in positive welfare indicators when they occupied the PPCC compared to the Penguinarium, although these changes did not follow the exact patterns we predicted. Instead of increasing their overall activity levels, the penguins shifted their activity from land to water by decreasing their time spent walking and spending more time swimming. We did see an increase in positive social behavior, and the penguins engaged in less agonistic behavior. Their behavior was generally consistent between study phases despite the variation in subjects and methods between the two phases. These consistent trends lend strong support to the hypothesis that the differences in behavior we observed were, in fact, linked to the two habitats in this study. Given that most of their behaviors were strongly influenced by seasonality, the results also highlight the importance of longitudinal data for understanding penguin welfare. Without conducting observations across the seasonal breeding cycle, we could have easily conflated differences based on season and habitat. We encountered these types of seasonal confounds while analyzing this dataset for patterns of behavior before, during, and after wearing TDRs [35]. Ultimately, moving to the larger, more complex PPCC appeared to have positive benefits for the welfare of these king penguins.

### 4.1. Changes in Aquatic Behaviors

Based on both TDR and observational data, the penguins showed a three to fourfold increase in time spent swimming in the PPCC compared to in the Penguinarium. Thus, there was a great deal of consistency between the two methods even though the TDRs sampled behavior 24 h a day, whereas we collected observational data from 08:00 to 16:00. In the Kerguelen Islands, king penguins rearing chicks spent about half their daylight hours in the water [26]. Although the penguins in this study did not spend as much time in the water as these free-ranging individuals, their energetic and foraging demands were different (e.g., few chicks reared during this study; readily available food sources outside of the water). Others have noted that captive penguins who are not fed in pools often spend little time foraging and more time resting on land [39]. When captive Humboldt penguins were given the opportunity to feed on live fish, their time spent swimming increased significantly [40]. The introduction of food-based enrichment devices also increased time spent swimming by captive Magellanic and southern rockhopper penguins [41]. In contrast, Kalafut and Kinley [42] used radio-frequency identification (RFID) technology to automatically log swimming by captive little penguins (*Eudyptula minor*) but did not observe increased swimming in the presence of a fish-based enrichment device. Regardless, feeding practices were consistent between habitats in this study and likely do not explain the increased amount of time spent swimming in the PPCC.

Because the king penguins in this study did not rely on the water to forage, their use of the pool likely reflected a different motivation. Seasonal swimming patterns observed in this study support the interpretation that the penguins swam due to an intrinsic drive. Like their wild counterparts, they swam the most during the non-breeding season [27], followed by the breeding season, with the least swimming occurring around molt [29]. Another factor that could have encouraged swimming was the complexity of the PPCC pool. Humboldt penguins increased their time swimming after non-food-based enrichment was added to their pool in another study, although the post-enrichment results from that study were difficult to interpret due to the onset of molt [39]. In the PPCC, several of the penguins showed a preference for floating in the water column above the bubbler, which was one of several complex elements not available in the Penguinarium pool.

One factor that seems unlikely to have influenced pool use in this study was visitor presence. Although we did not systematically assess visitor numbers, we observed the penguins in both habitats during periods when they were closed to visitors, and swimming patterns remained unchanged. Similarly, Edes et al. [12] found no relationship between pool use and crowd size, crowd composition, or noise levels in king penguins living in another mixed-species zoo colony. Crowd size did not affect the pool use of captive Humboldt penguins, either [39].

Swimming trends could indicate that the larger, more complex pool in the PPCC was simply preferred by the penguins. Some individuals, such as King 23, Arthur, and Gertie, hardly swam at all in the Penguinarium, making the increase in swimming in the PPCC particularly dramatic. Lola, who chose not to swim in either habitat, suffered from medical problems that caused chronic lameness. Lola’s behavior shows how individual differences in age and health can impact behavioral choices, highlighting the importance of assessing welfare at the individual level. One strength of this study was our ability to track penguins on an individual basis. Zoo researchers were also able to track individual little penguins through automated logging devices, and they also observed a great deal of individual variation in time spent swimming. Overall rates of swimming were incredibly low for all of the little penguins, but they did show a preference for swimming in warmer water [42]. The temperature of the PPCC pool is colder than that of the Penguinarium, and we cannot rule out the temperature differences between the habitats as a factor shaping their preference.

The TDR data provided a window into the activities of the penguins throughout the day and night, which is important given the need to consider animal welfare on a 24 h timescale [43]. King penguins are considered somewhat crepuscular and are known to forage throughout the night [44], although they do so less frequently and at shallower depths than during daytime [26]. The TDR data showed that swimming in this study coincided with day-phase lighting in both habitats, suggesting these penguins did not utilize the pool in darkness. Half of the TDR data were collected in June, when the penguins’ artificial day length is at its maximum and the habitat is light from 03:45 to 22:15. With no need to forage at night, the penguins may have had no need to enter the pool in darkness. Similarly, captive little penguins were less willing to dive for fish in their pool under darker conditions, although they did still swim at the surface [45]. The light schedule was the same in both habitats in this study, so it is not entirely clear why the penguins tended to stop swimming earlier in the evening in the PPCC than in the Penguinarium. Perhaps the reduced competition for space in the larger PPCC pool gave the penguins more ability to follow their internal schedules. This could reflect an improved welfare state. There are few published data showing hourly swimming patterns in captive penguins; Humboldt penguins seemed to swim equally throughout the hours of the day in one study, but those observations were limited to the hours when the zoo was open [40], rather than capturing the full activity cycle.

The design of the pool in the PPCC also gave the penguins more choices, including swimming depth. When given the opportunity, the penguins spent less time swimming at the water surface and increased their time swimming at depths greater than 2 m, suggesting that deeper pools are preferred by this larger penguin species. However, they did not spend much time swimming below 6 m, suggesting that the pool floor was not an appealing location for them. The penguins also engaged in more bathing behaviors in the PPCC, and they tended to perform these preening and adjusting movements more during the non-breeding season, when they were already swimming more. The narrow Penguinarium pool likely made these behaviors difficult to perform for these large birds. Together, these data on pool usage show how a POE study design can provide valuable information for the design of future habitats. Given the choice, king penguins seem to prefer an open water surface and depths extending to 6 m.

### 4.2. Changes in Other Solitary Behaviors

On land, the king penguins showed notable differences in locomotor and inactive behaviors between the two habitats. The most frequent behavior we observed was standing, which is consistent with penguins in other studies [17,46,47]. Inactivity is also the most common behavior of wild king penguins during the breeding season [44]. The penguins spent significantly less time walking in the PPCC compared to in the Penguinarium. Visually, their activity budget showed an inverse relationship between swimming and walking based on habitat. It seems as if their overall activity level remained the same, whereas the preferred venue—land or water—changed with the habitat. In the Penguinarium, the penguins tended to form a line and continuously walk around the perimeter of the circular land space, a behavior that was not as pronounced in the larger PPCC. This trend could have been related to differences in the land shape between the two habitats. It may also suggest an overall motivation to perform a certain amount of active behavior, with a preference for swimming expressed by the increase in this behavior in the larger pool space. Walking behavior also showed a seasonal effect, with less walking during molt (when the penguins tended to stand in place) and more during the breeding season. The increased walking during the breeding season likely reflected species-typical behaviors related to mate and breeding site selection.

The penguins also showed changes in their preferred inactive posture in the PPCC. Although we felt that it was redundant to run the GLMM on both standing and laying, the increase in laying in the PPCC seems to have been accompanied by a decrease in standing. One possible explanation is a preference for laying in “snow” (from the ice machine) that emerged in the PPCC. The penguins tended to congregate directly underneath the ice machine, sometimes allowing it to gradually bury them in snow. Furthermore, they increased their time spent laying in the Penguinarium during the second phase of the study, when the Penguinarium also contained an ice machine. Given the differences in subjects and data collected between the two phases, we cannot definitively say that snow caused the increased laying, but it seems likely. Another study also found that adding crushed ice to a king penguin habitat increased time resting, but it also precipitated an increase in agonistic behaviors due to competition for the ice [48]. Although ice did not appear to increase agonism in this study, together these findings indicate that ice is a highly preferred resource for king penguins.

Changes in other solitary behaviors were minimal. Time spent preening and adjusting were similar between the two habitats. Although there was a slight increase in feeding in the PPCC, we rarely observed this behavior. Fish were quickly consumed whole after being fed by hand or in pans on land, or broadcast-fed in the water. These short bouts of consumption were difficult to capture in interval data. It is possible that the king penguins were able to take greater advantage of food offered in the pool when they spent more time swimming. Feeding was one of the few behaviors that showed a significant sex difference in this study, with females spending more time feeding. Although wild king penguins also show sex differences in foraging behaviors, males spend more time incubating than females, followed by longer foraging trips to recoup their greater loss of body fat [49]. Time spent investigating and performing other rarer behaviors (which we lumped as “other”) did not occur frequently enough to analyze statistically. Taken together, these results suggest that the primary habitat design factors shaping changes to the king penguin activity budget between the two spaces were largely shaped by opportunities to swim and walk on land.

### 4.3. Changes in Social Dynamics

Changes in social behaviors between the two habitats were more nuanced than locomotor trends. Positive social behaviors (both allopreening and displaying) increased in the PPCC in Phase 1, supporting our initial prediction. However, the change in allopreening was not sustained in Phase 2, and the penguins displayed more in the Penguinarium in Phase 2. These behaviors were both fairly rare, and it is difficult to determine whether these trends were due to differences in the composition of study subjects or another factor. Males in this study displayed more than females, showing the opposite pattern to captive Humboldt penguins [50]. Surprisingly, the mixed model showed fewer displays overall during the breeding season compared to the nonbreeding season, with the highest rate of displays occurring during molt. For the sake of data analysis, we classified two full months as the molt season, and individuals molted at various times throughout this range. Given that individuals vary in the start date and duration of their molt, it is possible that individuals who underwent earlier or shorter molts transitioned to breeding behaviors earlier. King penguins who breed earlier in the wild tend to be more successful [30], and perhaps there is a stronger behavioral drive for those breeding early.

It is possible that differences in visibility between the two habitats might explain some of these changes, rather than meaningful changes in the behavior of the penguins. In Phase 1, the king penguins tended to congregate behind an outcropping extending into the central portion of the habitat from their preferred side (the east side) of the habitat during the breeding season. The animal care staff referred to this area as “lover’s lane”, and it was largely not visible to observers. Increases in social behaviors observed in the PPCC could reflect greater visibility of the king penguins in the more open habitat space. Visibility could also explain why displaying differed between study phases. Modifications made to the Penguinarium prior to the penguins’ 2019 return—specifically, increased access to the central area of the habitat—were intended to create a more open habitat, resulting in better visibility for observers.

Overall rates of agonistic behavior were highest during the breeding season in both habitats but were higher in the Penguinarium compared to in the PPCC. This trend was consistent for both study phases and across both agonistic directionalities (given and received). In Phase 1, animal care staff sometimes provided the king penguins with access to an additional space, which observers could not see, to reduce aggression during the breeding season. Availability of space may also have modulated proximity patterns, resulting in reductions in agonistic behaviors, as has been observed for Hanuman langurs (*Presbytis entellus*) [51]. Following this aforementioned modification to the Penguinarium, time spent alone no longer significantly differed between the two habitats. Subjectively, it is also possible that the decrease in walking in the PPCC was also related to the decrease in agonistic behavior, as inter-specific agonistic encounters were often observed as the king penguins walked the perimeter of the Penguinarium.

The fact that we saw more agonistic behavior in the Penguinarium despite these visibility challenges and mitigation strategies suggests that this trend can be attributed to habitat design. Furthermore, wild king penguins engage in more agonistic behaviors during chick rearing than incubation [31,44]. Chicks were present in both habitats in Phase 2 of the study, but the highest rates of agonistic behavior occurred in the Penguinarium in Phase 1. Besides breeding stage, territory location is the primary predictor of aggressive behavior in wild king penguins. As previously noted, the central positions in breeding colonies are preferred, leading to increased aggression from birds in the crowded colony centers as individuals defend priority nest locations [31]. Together, these lines of evidence support the interpretation that differences in social density related to habitat design affected agonistic behavior in this study.

Regardless of habitat, the king penguins spent most of their time in proximity to other king penguins, followed by time alone, with the least time spent in proximity to allospecifics. The observed tendency to congregate near other conspecifics is consistent with data from a mixed colony of chinstrap and gentoo penguins, who synchronized their behavior into species-specific patterns within the same habitat space [46]. The king penguins discussed here spent less time near both conspecifics and allospecifics in the PPCC. However, only time spent near other king penguins showed a seasonal trend, with less time in proximity during the breeding and molting seasons. The trend to spend less time near other king penguins during the breeding season is counterintuitive. As with display behavior, this unexpected result could reflect individual differences in the timing of the breeding and molting seasons that were not captured by the broad ranges we used to define seasons in data analysis.

Proximity trends may also be related to variation in colony composition over this longitudinal study. When the penguins occupied the Penguinarium in Phase 1, the colony contained only three gentoo penguins. An additional 20 gentoos were added to the group after the initial move to the PPCC, but overall social density may not have increased that much due to the size of the PPCC habitat. Thus, proximity to other species did not differ statistically between habitats in Phase 1. However, proximity to other species was significantly higher in Phase 2 after the larger colony moved to the Penguinarium. The mixed model did not identify any demographic factors, such as age or sex, that explained social proximity trends. In fact, most behaviors we observed did not show variation along these dimensions. King penguins have previously been reported by animal keepers to show no sex-based differences in personality, unlike other penguin species [52].

Individual differences also played a critical role in shaping proximity patterns. Some individuals consistently changed their proximity both to king penguins and other species between the two habitats, whereas other individuals showed opposite trends depending on the identity of the species in proximity. In one study, aquarium-housed Humboldt penguins showed significant variation in how much time different pairs spent in proximity to their mates [47]. One of the limitations of this study is that we did not identify the individuals to whom focal penguins were in proximity, so we cannot comment on how their social networks or pair bonds could have affected proximity patterns. Generally, there seem to be fewer studies on penguin social behavior in captive settings, and generating more information about their complex social lives could enhance their welfare in the care of humans.

## 5. Conclusions: Implications for Habitat Design and Penguin Welfare

By definition, a POE is based on the behavior of a single group of animals moving between enclosures, typically within a single institution [1]. As a result, it is difficult to differentiate between individual, group-level, and species trends, and to rule out changing social dynamics or the passage of time as confounding factors. By including multiple moves between the same enclosures, this study has a unique POE design that more strongly links the observed behavioral changes to habitat design. Yet, because the PPCC is both substantially larger and more complex than the Penguinarium, it is still not possible to tease apart the relative contributions of habitat size and complexity. A study at the Dallas Zoo attempted to differentiate these factors by examining the behavior of one group of African elephants (*Loxodonta africana*) in three habitat spaces that varied along a continuum of size and complexity [53]. However, their results varied, with behaviors like foraging showing a potentially greater impact of complexity, whereas stereotypy was influenced more by habitat size [53]. A multi-institutional study design is likely needed to determine how space and complexity differentially affect animal welfare.

Although a POE for one group cannot identify species trends, welfare is experienced at the individual level, and a POE can say a lot about what individual animals want. Recently, Dawkins [54] advanced a new definition of animal welfare, describing it simply as “health and what animals want”. This definition stands in opposition to affect-based models of animal welfare, e.g., [6], which Dawkins argues are too challenging to operationalize given the current limitations in scientifically measuring animal emotion. By Dawkins’s definition, giving animals what they want should improve their welfare. Thus, incorporating preferred features into habitat design is likely to generate the greatest benefits for animal welfare [55]. However, giving animals what they want depends on conducting studies like these across varied contexts in order to establish ideal habitat design and management processes.

## Figures and Tables

**Table 1:** Study phases and sub-periods for moves between the Penguinarium and Polk Penguin Conservation Center (PPCC). TDR = time–depth recorder.

Study Phase	Sub-Period	Data Collected: Hours (Number of Observations)	Habitat	Dates	Description
Phase 1 2015–2017	Penguinarium I	223.3 (1476)	Penguinarium	2 March 2015–29 February 2016	Regular data collection prior to the first move
				8 June 2015–24 July 2015	TDR data collection during the breeding season
				4 December 2015–19 January 2016	TDR data collection during the non-breeding season
	Interlude	0 (0)	Penguinarium	1 March 2016–12 May 2016	King penguins not observed during their molt in the Penguinarium
	PPCC II	321.4 (2069)	PPCC	13 May 2016–31 July 2017	Regular data collection after their first move to the PPCC
				6 December 2016–18 January 2017	TDR data collection during the non-breeding season
				8 June 2017–23 July 2017	TDR data collection during the breeding season
No data collection		0 (0)	PPCC	1 August 2017–4 August 2019	Penguins not observed
Phase 2 2019–2022	PPCC III	8.4 (103)	PPCC	5 August 2019–6 September 2019	Regular data collection
	Penguinarium IV	88.6 (1078)	Penguinarium	10 September 2019–28 June 2021	Penguins not viewable by the public. Note that this period and the one below overlap, as the king penguins were moved gradually to the PPCC as they completed their annual molt.
	PPCC V	53.1 (642)	PPCC	10 June 2021–29 April 2022	Penguins not viewable by the public until building re-opening on 14 February 2022

**Table 2:** Subject information for king penguin habitat change study. M = male; F = female. Names marked with an * indicate penguins that were studied through both study phases, 2015–2017 and 2019–2022.

Name	Sex	Age at Observation Onset	Study Periods
Arthur	M	2.62	III–V
Gertie	F	3.68	III–V
King 22 *	M	13.49	I–V (all)
King 23	M	13.48	I and II
King 25 *	F	14.01	I–V (all)
King 26	F	4.92	I and II
King 77 *	F	26.92	I–V (all)
Kong *	M	19.58	I–V (all)
Lola	F	26.97	I and II
Slim	M	21.60	I and II

**Table 3:** Penguin ethogram. Behaviors marked * were scored both on intervals and as all-occurrence behaviors.

Behavior	Operational Definition
Activity (Intervals/Scans)	
Allopreen *	Rubbing the head, bill, or flipper on another individual, or “mouthing” another individual
Receive allopreen *	Receiving preening from another penguin
Contact aggression *	Pecking, wing blows, or gripping and twisting with bill directed at another penguin
Receive contact aggression *	Receiving contact aggression from another penguin
Noncontact aggression *	Lunging at another penguin or attempting to peck one without actually making contact, or pointing the head toward and aggressively vocalizing at another penguin
Receive noncontact aggression *	The focal penguin is the recipient of lunging, attempted pecks, or aggressive vocal displays from another penguin.
Display	Performing an ecstatic or mutual display
Mate *	Any breeding behavior between penguins, including dorso-ventral mounting and cloacal rubbing; also includes rapidly moving flippers (“vibrating”) on the body of another
Chick-directed	Any behavior directed toward a chick, including regurgitating food, preening chicks, and restraining chicks from moving
Keeper-directed	Following, pointing, leaning towards (>45°) and vocalizing at or otherwise interacting with a human (keeper) in the exhibit; includes breeding behaviors directed towards a keeper, e.g., flipper vibrating
Investigate	Pecking at or manipulating rockwork or objects on land or in the pool, including enrichment items, ice cubes, whale bones on land, bubbles and floating debris in the pool, kelp, underwater decorations, or shaved ice/snow
Bathe	Preening or adjustment movements in water, including tail wags and shaking movements; can occur in the pool or using water features (fountains, etc.) on land
Surface swim	Penguin uses the water to propel itself at a depth less than 1 m, including porpoising or floating; does not include walking on submerged land
Dive	Swimming underwater at depths greater than 1 m
Walk	Moving at any pace by lifting and setting down each foot in turn; includes using feet on the ground to propel the body across submerged land
Vocalize	Producing sounds using the mouth; bill is open
Feed	Ingesting or chewing food or drinking water; includes being fed by keeper and manipulating food in the water (even if ingestion is not observed)
Shiver	Slightly shaking the whole body; less intense than adjustment movements and involves the whole body instead of just the flippers (flipper vibrating is a component of mating behavior)
Preen	Includes contact between the bill and feathers, rubbing the head over another body part, wing rub on head or neck, or scratching with feet
Adjust	Shaking and stretching movements, including head shake, head bob, body shake, tail wag, or rapid wing flap; may perform these behaviors in the water as part of a bathing bout
Stand	Resting in an upright position; may be moving the head or eyes to observe the surroundings, or head may be tucked under the wing while the penguin is sleeping
Lay	Resting on the belly or in any position other than standing
Other	Any other behavior not detailed elsewhere involving vigorous movement; may include shaking the head in the nest (looks like a partial ecstatic display) or making biting movements in the air
Not visible	Behavior cannot be definitively determined and/or the observer has no idea where the penguin is located
Additional All-Occurrence Behaviors	
Ecstatic display	A penguin is standing up on its toes, flippers extended vertically, head and bill also held up vertically, and vocalizing; in king penguins, the display involves tilting the head back, trumpeting, and then bowing; may also involve rapid bill movements (“clacking”) in kings
Mutual display	Two penguins in proximity to one another simultaneously performing an ecstatic display, or two penguins (usually kings) rubbing their beaks together or “kissing”
Porpoise	Leaping in and out of the water in short, shallow arcs; must include two leaps in quick succession to count as a porpoising bout
Toboggan	Horizontal movement on land by sliding on the belly, often using the feet or flippers to propel the body forward
Land bathe	Preening or adjustment movements including tail wags and shaking movements occurring in land-based water features such as the splash zone, waterfalls, or hose jets
Water bathe	Preening or adjustment movements including tail wags and shaking movements occurring when the penguin is in the pool or on submerged land

**Table 4:** Wilcoxon two-sample with Monte Carlo exact test results comparing TDR data between the Penguinarium and the Polk Penguin Conservation Center. Significant differences (Pr > |S-Mean| of 0.05 or below) are indicated with an asterisk (*), Pr > |S-Mean| of 0.01 or below are indicated with a double asterisk (**), and Pr > |S-Mean| of <0.001 are indicated with a triple asterisk (***).

Comparison	Average ± SE	Statistic (S)	Z	Pr > |Z|	Pr > |S-Mean|
Percentage of time between habitats *	Peng.: 5.44 ± 0.97% PPCC: 17.71 ± 1.28%	49.00	−1.9954	0.0460	0.0494
Surface by habitat **	Peng.: 69.31 ± 3.12% PPCC: 40.06 ± 3.00%	166.00	−2.9355	0.0032	0.0024
0–2 m by habitat	Peng.: 30.69 ± 3.12% PPCC: 24.92 ± 2.95%	220.00	−0.7934	0.4276	0.4373
2–4 m by habitat ***	Peng.: -- PPCC: 27.59 ± 0.49%	344.00	4.5829	<0.0001	<0.0001
4–6 m by habitat ***	Peng.: -- PPCC: 7.37 ± 0.07%	344.00	4.5829	<0.0001	<0.0001
6–8 m by habitat ***	Peng.: -- PPCC: 0.06 ± 0.00%	320.00	3.8083	0.0001	<0.0001
Average depth between habitats ***	Peng.: 0.06 ± 0.01 m PPCC: 1.43 ± 0.03 m	332.00	3.6495	0.0005	0.0001

**Table 5:** Wilcoxon two-sample with Monte Carlo exact test results for comparisons of behavioral variables between the Penguinarium (Peng) and the Polk Penguin Conservation Center (PPCC) by study phase. Significant differences (Pr > |S-Mean| of 0.05 or below) are indicated with an asterisk (*), Pr > |S-Mean| of 0.01 or below are indicated with a double asterisk (**), and Pr > |S-Mean| of <0.001 are indicated with a triple asterisk (***).

Behavior	Average ± SE	Statistic (S)	Z	Pr > |Z|	Pr > |S-Mean|
	Interval Behaviors				
Swim Phase 1 ***	Peng.: 7.63 ± 3.13% PPCC: 24.65 ± 5.19%	2,333,269	−13.4543	<0.0001	<0.0001
Swim Phase 2 ***	Peng.: 0.89 ± 1.29% PPCC: 12.03 ± 5.21%	728,818	10.3590	<0.0001	<0.0001
Feed Phase 1 ***	Peng.: 0.16 ± 0.19% PPCC: 0.29 ± 0.32%	2,333,269	−13.4543	<0.0001	<0.0001
Feed Phase 2 ***	Peng.: 0.22 ± 0.36% PPCC: 0.38 ± 0.52%	728,818	10.3590	<0.0001	<0.0001
Lay Phase 1***	Peng.: 2.36 ± 1.73% PPCC.: 3.77 ± 2.01%	2,561,076	−4.6298	<0.0001	<0.0001
Lay Phase 2 **	Peng.: 3.92 ± 3.06% PPCC: 6.56 ± 3.92%	691,578	2.6400	0.0042	0.0076
Walk Phase 1 ***	Peng.: 18.63 ± 3.13% PPCC: 6.88 ± 1.56%	3,014,571	14.8676	<0.0001	<0.0001
Walk Phase 2 ***	Peng.: 19.05 ± 4.47% PPCC: 5.84 ± 2.45%	564,435	−12.3639	<0.0001	<0.0001
	All-Occurrence Behaviors				
Displays Phase 1 ***	Peng.: 0.39 ± 0.46 PPCC: 0.70 ± 0.55	2,582,905	−3.6770	0.0002	0.0002
Displays Phase 2 *	Peng.: 0.88 ± 0.88 PPCC: 0.71 ± 1.05	673,110.5	−2.0448	0.0409	0.0494
Bathing Phase 1 ***	Peng.: 0.00 ± 0.00 PPCC: 0.85 ± 0.62	2,532,078	−9.2052	<0.0001	<0.0001
Bathing Phase 2 ***	Peng.: 0.28 ± 0.59 PPCC: 1.44 ± 1.26	696,364	5.4141	<0.0001	<0.0001
Agonism—given Phase 1 ***	Peng.: 3.59 ± 1.23 PPCC: 1.37 ± 0.63	2,784,090	8.6045	<0.0001	<0.0001
Agonism—given Phase 2 *	Peng.: 4.95 ± 1.68 PPCC: 3.61 ± 1.33	666,041	−2.1660	0.0303	0.0309
Agonism—received Phase 1 ***	Peng.: 3.01 ± 1.00 PPCC: 1.05 ± 0.53	2,805,129	10.2069	<0.0001	<0.0001
Agonism—received Phase 2 *	Peng.: 2.08 ± 1.30 PPCC: 1.51 ± 1.12	667,904.5	−2.1100	0.0175	0.0335
Agonism total Phase 1***	Peng.: 6.60 ± 1.95 PPCC: 2.42 ± 1.03	2,875,473	11.6583	<0.0001	<0.0001
Agonism total Phase 2 **	Peng.: 4.95 ± 2.56 PPCC: 3.61 ± 2.19	658,138	−3.0385	0.0024	0.0022
Allopreening Phase 1 **	Peng.: 0.22 ± 0.26 PPCC: 0.43 ± 0.36	2,593,365	−2.8504	0.0044	0.0041
Allopreening Phase 2	Peng.: 0.28 ± 0.48 PPCC: 0.25 ± 0.38	680,505	0.4882	0.6254	0.6650
	Proximity Measures				
Alone Phase 1 *	Peng.: 46.72 ± 4.40% PPCC: 44.03 ± 4.67%	2,681,066	2.1504	0.0315	0.0307
Alone Phase 2	Peng.: 34.77 ± 6.01% PPCC: 38.57 ± 6.96%	687,145	0.7278	0.4668	0.4676
Proximate to another king Phase 1	Peng.: 45.70 ± 4.36% PPCC: 46.94 ± 4.66%	2,572,424	−1.4941	0.1352	0.1361
Proximate to another king Phase 2	Peng.: 56.66 ± 6.19% PPCC: 58.92 ± 7.05%	690,250.5	1.0125	0.3113	0.3132
Proximate to individual of another species Phase 1	Peng.: 7.44 ± 1.64% PPCC: 8.35 ± 1.96%	2,625,542	0.3365	0.7365	0.7344
Proximate to individual of another species Phase 2 ***	Peng.: 8.48 ± 2.84% PPCC: 2.51 ± 1.72%	604,540	−9.9842	<0.0001	<0.0001

**Table 6:** Wilcoxon two-sample with Monte Carlo exact test results for comparisons of behavioral variables between the Penguinarium (Peng) and the Polk Penguin Conservation Center (PPCC). Significant differences (Pr > |S-Mean| of 0.05 or below) are indicated with an asterisk (*), Pr > |S-Mean| of 0.01 or below are indicated with a double asterisk (**), and Pr > |S-Mean| of <0.001 are indicated with a triple asterisk (***).

Behavior	Average ± SE	Statistic (S)	Z	Pr > |Z|	Pr > |S-Mean|
	Interval Behaviors				
Swim ***	Peng.: 4.78 ± 2.00% PPCC: 21.31 ± 3.95%	6,205,560	−18.1117	<0.0001	<0.0001
Feed	Peng.: 0.19 ± 0.18% PPCC: 0.31 ± 0.27%	6,835,768	−1.7506	0.0800	0.0805
Lay ***	Peng.: 3.02 ± 1.59% PPCC: 4.50 ± 1.84%	6,738,055	−5.1233	<0.0001	<0.0001
Walk ***	Peng.: 18.81 ± 2.58% PPCC: 6.60 ± 1.31%	7,702,521	17.0888	<0.0001	<0.0001
	All-Occurrence Behaviors				
Displays *	Peng.: 0.60 ± 0.44 PPCC: 0.70 ± 0.50	6,820,722	−2.0932	0.0363	0.0397
Bathing ***	Peng.: 0.12 ± 0.23 PPCC: 1.01 ± 0.58	6,675,406	−10.6635	<0.0001	<0.0001
Agonism—given ***	Peng.: 3.29 ± 0.99 PPCC: 1.57 ± 0.60	7,115,008	7.3697	<0.0001	<0.0001
Agonism—received ***	Peng.: 2.62 ± 0.79 PPCC: 1.17 ± 0.50	7,135,458	8.5281	<0.0001	<0.0001
Agonism total ***	Peng.: 5.91 ± 1.55 PPCC: 2.74 ± 0.98	7,256,697	9.9999	<0.0001	<0.0001
Allopreening **	Peng.: 0.25 ± 0.24 PPCC: 0.39 ± 0.27	6,810,070	−3.2308	0.0012	0.0010
	Proximity Measures				
Alone *	Peng.: 41.68 ± 3.60% PPCC: 42.59 ± 3.86%	6,722,278	−2.3989	0.0164	0.0166
Proximate to another king ***	Peng.: 50.33 ± 3.62% PPCC: 50.11 ± 3.90%	6,616,997	−4.2561	<0.0001	<0.0001
Proximate to individual of another species **	Peng.: 7.88 ± 1.49% PPCC: 6.80 ± 1.47%	6,985,366	2.8379	0.0023	0.0044

**Table 7:** Generalized linear mixed model (GLMM) results for behavioral variables. The Penguinarium is abbreviated as Peng. and the Polk Penguin Conservation Center is abbreviated as PPCC. Significant differences (Pr > |t| of 0.05 or below) are indicated with an asterisk (*), Pr > |t| of 0.01 or below are indicated with a double asterisk (**), and Pr > |t| of <0.001 are indicated with a triple asterisk (***).

Behavior	Effect		Estimate	S.E.	DF	T Value	Pr > |t|	Lower	Upper
Interval Behaviors									
Swim	Intercept **		−2.5467	0.6182	9	−4.12	0.0026	−3.9453	−1.1482
	Habitat ***	PPCC	1.5279	0.0925	5355	16.53	<0.0001	1.3466	1.7091
	Habitat	Peng.	0.0000	.	.	.	.	.	.
	Season	Breeding	−0.1351	0.0999	5355	−1.35	0.1762	−0.3309	0.0607
	Season ***	Molting	−0.7875	−0.788	5355	−6.19	<0.0001	−1.0369	−0.5381
	Season	Other	0.0000	.	.	.	.	.	.
Lay	Intercept ***		−2.9178	0.4356	9	−6.70	<0.0001	−3.9031	−1.9324
	Habitat ***	PPCC	1.0181	0.1641	5356	6.20	<0.0001	0.6963	1.3399
	Habitat	Peng.	0.0000	.	.	.	.	.	.
	Time of day ***	A.M.	−1.3525	0.1631	5356	−8.29	<0.0001	−1.6722	−1.0328
	Time of day	P.M.	0.0000	.	.	.	.	.	.
Feed	Intercept ***		−5.6780	0.3158	8	−17.98	<0.0001	−6.5063	−4.9497
	Habitat **	PPCC	0.6875	0.2653	5357	2.59	0.0096	0.1674	1.2076
	Habitat	Peng.	0.0000	.	.	.	.	.	.
	Sex *	Female	0.7156	0.3530	5357	2.03	0.0427	0.0237	1.4076
	Sex	Male	0.0000	.	.	.	.	.	.
Walk	Intercept ***		−0.7208	0.0945	9	−7.63	<0.0001	−0.9345	−0.5071
	Habitat ***	PPCC	−0.7913	0.0679	5352	−11.66	<0.0001	−0.9244	−0.6583
	Habitat	Peng.	0.0000	.	.	.	.	.	.
	Season ***	Breeding	0.3272	0.0670	5352	4.88	<0.0001	0.1959	0.4585
	Season	Molting	−0.0126	0.0783	5352	−0.16	0.8726	−0.166	0.1409
	Season	Other	0.0000	.	.	.	.	.	.
All-Occurrence Behaviors									
Bathe	Intercept ***		−5.7962	0.4530	9	−12.80	<0.0001	−6.8209	−4.7716
	Habitat ***	PPCC	2.6686	0.2891	5355	9.23	<0.0001	2.1019	3.2353
	Habitat	Peng.	0.0000	.	.	.	.	.	.
	Season ***	Breeding	−0.9195	0.2718	5355	−3.38	0.0007	−1.4523	−0.3867
	Season	Molting	−0.00789	0.3034	5355	−0.03	0.9792	−0.6026	0.5868
	Season	Other	0.0000	.	.	.	.	.	.
Display	Intercept ***		−3.1501	0.1787	8	−17.63	<0.0001	−3.5621	−2.7381
	Sex ***	Female	−0.8233	0.1928	5355	−4.27	<0.0001	−1.2014	−0.4453
	Sex	Male	0.0000	.	.	.	.	.	.
	Habitat	PPCC	0.3595	0.1950	5355	1.84	0.0652	−0.0227	0.7417
	Habitat	Peng.	0.0000	.	.	.	.	.	.
	Season ***	Breeding	−1.2276	0.2375	5355	−5.17	<0.0001	−1.6932	−0.7620
	Season *	Molting	0.5002	0.2363	5355	2.12	0.0343	0.0370	0.9634
	Season	Other	0.0000	.	.	.	.	.	.
Agonism	Intercept ***		−1.4374	0.1329	9	−10.81	<0.0001	−1.7381	−1.1367
	Habitat ***	PPCC	−0.6516	0.1275	5353	−5.11	<0.0001	−0.9015	−0.4017
	Habitat	Peng.	0.0000	.	.	.	.	.	.
	Season ***	Breeding	0.7832	0.1348	5353	5.81	<0.0001	0.5190	1.0474
	Season *	Molting	−0.3763	0.1625	5353	−2.32	0.0206	−0.6948	0.0577
	Season	Other	0.0000	.	.	.	.	.	.
Proximity Measures									
Alone	Intercept		0.1559	0.1254	9	1.24	0.2453	−0.1278	0.4396
	Habitat ***	PPCC	0.2638	0.0365	5352	7.23	<0.0001	0.0923	0.3353
	Habitat	Peng.	0.0000	.	.	.	.	.	.
	Season ***	Breeding	0.1742	0.0422	5352	4.13	<0.0001	0.0916	0.2569
	Season ***	Molting	0.2603	0.0470	5352	5.54	<0.0001	0.1682	0.3523
	Season	Other	0.0000	.	.	.	.	.	.
	Time of day ***	A.M.	−0.090	0.0243	5352	−3.71	0.0002	−0.1378	−0.0425
	Time of day	P.M.	0.0000	.	.	.	.	.	.
King	Intercept ***		1.4124	0.2123	9	6.65	<0.0001	0.9321	1.8928
	Age ***		−0.06069	0.0075	5352	−8.12	<0.0001	−0.075	−0.0460
	Habitat ***	PPCC	−0.1451	0.0317	5352	−4.58	<0.0001	−0.2073	−0.0830
	Habitat	Peng.	0.0000	.	.	.	.	.	.
	Season ***	Breeding	−0.1833	0.0371	5352	−4.94	<0.0001	−0.2561	−0.1105
	Season ***	Molting	−0.1865	0.0417	5352	−4.48	<0.0001	−0.2682	−0.1048
	Season	Other	0.0000	.	.	.	.	.	.
Other	Intercept ***		−4.3134	0.2003	9	−21.54	<0.0001	−4.7665	−3.8603
	Habitat ***	PPCC	−1.0387	0.2600	5357	−3.99	<0.0001	−1.5484	−0.5289
	Habitat	Peng.	0.0000	.	.	.	.	.	.

## Data Availability

Data are available from the corresponding author upon reasonable request.
